# Development of a training program prototype to enhance implementation leadership competencies and behaviours of Chinese unit nurse managers: a qualitative descriptive study

**DOI:** 10.1186/s12912-024-01989-8

**Published:** 2024-05-30

**Authors:** Wenjun Chen, Ian D. Graham, Jiale Hu, Krystina B. Lewis, Junqiang Zhao, Wendy Gifford

**Affiliations:** 1 Xiangya School of Nursing, Central South University, 172 Tongzipo Road, Changsha, Hunan 410013 China; 2 School of Nursing, Faculty of Health Science, University of Ottawa, Ottawa, ON Canada; 3 Center for Research on Health and Nursing, University of Ottawa, Ottawa, ON Canada; 4 School of Epidemiology and Public Health, Faculty of Medicine, University of Ottawa, Ottawa, ON Canada; 5 Ottawa Hospital Research Institute, University of Ottawa, Ottawa, ON Canada; 6 Department of Nurse Anesthesia, Virginia Commonwealth University, Richmond, VA USA; 7 Waypoint Research Institute, Waypoint Centre for Mental Health Care, Penetanguishene, ON Canada

**Keywords:** Evidence-based practice, Implementation science, Integrated knowledge translation, Nursing leadership, Qualitative descriptive

## Abstract

**Background:**

Unit nurse managers hold essential positions that can facilitate implementation of evidence-based practice. Studies showed that nurse managers in China lacked competencies and behaviours necessary to lead evidence-based practice implementation. The aim of the current study was to develop a context-fit training program prototype to enhance leadership competencies and behaviours regarding evidence-based practice implementation of Chinese unit nurse managers.

**Method:**

We used a descriptive qualitative study design and followed the integrated knowledge translation approach to co-develop the prototype in a tertiary hospital in Changsha, China. Seven nurse managers from the participated hospital and a researcher co-developed the prototype based on the Ottawa Model of Implementation Leadership (O-MILe). The development process encompassed four phases from November 2021 to March 2022 that involved group discussions (*n* = 4) and individual interviews (*n* = 21). All data were analysed by two independent researchers using the thematic analysis method.

**Results:**

Managers agreed that all O-MILe behaviours were important to evidence-based practice implementation, and only minor modifications were needed for clarification and adaptation. The actions managers identified that could operationalize the leadership behaviours were related to current clinical practices, evidence-based practice, nurses, patients, interprofessional staff members, incentives and resources, organization and external entities. Three types of general competencies related to evidence-based practice, professional nursing, and implementation leadership were identified. Multimodal activities such as lectures, experience sharing, group discussions, plan development and coaching were suggested to deliver the training program.

**Conclusions:**

All O-MILe leadership behaviours were perceived as essential for unit nurse managers to lead EBP implementation in the hospital context in China. We identified the leadership actions and the competencies required for nursing managers to implement EBP in China. Further studies are required to evaluate the acceptability and impact of this prototype. Further studies with large sample sizes across various clinical settings are needed to facilitate the generalization of the findings and gain an in-depth understanding of the program.

**Supplementary Information:**

The online version contains supplementary material available at 10.1186/s12912-024-01989-8.

## Introduction

Evidence-based practice (EBP) refers to practitioners’ conscientious, explicit and judicious use of current best evidence when making decisions about patient care [1]. The implementation of EBP is well-recognised as a global priority for bridging the gap between knowledge and action to reduce health inequities [2]. Researchers have found numerous factors that impede or enhance the implementation process, such as the quality of evidence being used, the context of implementation (e.g. healthcare systems or organizational settings) and the characteristics of individuals involved in the process [3].

Leadership is increasingly recognised as a strong factor for the implementation of EBP [4]. Leadership has been defined in many ways. A widely employed definition of leadership is that it is a process that influences others to accomplish common goals [5]. Unit-level nurse managers (hereafter referred to as unit nurse managers) oversee point of care nurses in healthcare organisations and are supervised by senior managers (e.g. nursing administrators, executives and directors); they are thus in a unique position to promote the implementation of EBP in their organisations [6, 7]. When proactively engaged, unit nurse managers’ leadership behaviours can promote the frontline use of research evidence and influence senior managers’ directives for implementing EBP [6].

Notably, a lack of leadership competencies and behaviours has been consistently identified as a prominent barrier to EBP implementation [4]. Due to the accumulating interest in leadership and EBP over the last decade, several researchers have developed and tested interventions to improve nursing leadership for EBP implementation. Kvist et al. [8] and Wallen et al. [9] developed such interventions and produced positive but non-statistically significant effects on outcomes such as leadership knowledge, belief in EBP, organisational readiness and culture that support EBP. Richter et al. [10] developed a generic implementation leadership intervention for healthcare managers and discovered that the intervention improved managers’ knowledge of leading EBP implementation and mixed effects on their behaviours change. Gifford et al. [11] developed a leadership-focused intervention for nurse managers to facilitate nurses implementing EBP. Authors found that the intervention had a positive influence on nurses’ use of evidence-based diabetic foot ulcer care practices in home care settings [12]. This intervention was guided by the Ottawa Model of Implementation Leadership (O-MILe), which provides a theoretical foundation for developing and operationalizing unit-level leadership to implement EBP in health care settings, identifying both the knowledge and skills required to develop implementation leadership and the specific behaviours required for practice [13]. In the O-MILe, Gifford and colleagues [13] specified that unit-level leaders could practice relations-oriented, change-oriented, and task-oriented implementation leadership behaviours to promote staff to implement EBP, meanwhile creating a positive environment and supportive organizational structures and procedures for the implementation process. According to Gifford et al. [4], implementation leadership is a multidimensional process of leading staff to conduct EBP implementation in clinical practice. In the process, leaders engage in various behaviours and activities to influence individuals, their environment, and organizational infrastructures to achieve optimal nursing practice and patient outcomes.

In China, nurse managers work at either the senior (i.e. nursing directors and sector nurse managers) or unit level (i.e. unit nurse managers) [14]. Nursing directors are responsible for the management of all nursing units in the hospital. They make budgetary decisions and organise and approve continuing education for nurses. Sector nurse managers are supervised by nursing directors and are responsible for the management of multiple units in their sectors (e.g. surgical, internal medicine, outpatient), with one or more unit nurse managers reporting to them. Unit nurse managers directly supervise point-of-care nurses and all activities related to the nursing practice as well as overseeing patient care in their respective units [15]. In China, unit nurse managers are familiar with clinical practice as they are all nurses with rich clinical nursing experiences [15]. One survey of 1300 nurse managers revealed that unit nurse managers communicated quite frequently with patients and their families to understand patients’ conditions [14]. They also influenced physicians’ support of nursing practice in multiple ways, such as coordinating nurse-physician relationships [14]. In addition, the Chinese work culture emphasizes respect for hierarchy and harmony with others [15, 16]. Leaders in formal management positions in China often have the authority and perceived superiority to command obedience and loyalty of subordinates within hierarchical working environments [15]. Specifically, according to Cheng et al. [15], leaders may assert their authority over subordinates and expect obedience. In summary, unit nurse managers in China hold essential leadership positions that can initiate and facilitate EBP implementation as they are familiar with current nursing practice and have perceived authority to directly influence nurses’ implementation of EBP and other key stakeholders in the process.

A scoping review found that leadership support is the strongest facilitator to implement EBP in the Chinese nursing context [2]. However, the results of several studies revealed that nurse managers lack the knowledge and skills necessary to lead EBP implementation [15, 17, 18]. Thus, interventions are needed to develop nursing managers’ implementation leadership competencies. In 2016, a group of international scholars developed a theory-informed leadership intervention to implement evidence-based pain management practices in Shanghai, China [19]. To date, this is the only intervention in China that focuses on the role of nursing leadership in promoting EBP implementation. The intervention focused on the leadership behaviours in the O-MILe. However, the researchers did not explore whether the implementation leadership behaviours specified in the O-MILe were essential for Chinese nurse managers, or whether the Chinese culture and nursing context influenced managers’ implementation leadership practices. Specifically, it is unknown whether the hierarchical Chinese work environment or the formal authority and position of nurse managers influences the types of leadership behaviours required to implement EBP. Also, the actions that managers need to take to lead EBP implementation in China remain to be determined. Therefore, there is a need to identify leadership behaviours that are essential for nurse managers to promote implementation of EBP in China, and to develop interventions that strengthen the competencies required for nursing managers to enact implementation leadership behaviours.

Implementation leadership competencies refer to the leadership knowledge, skills and abilities required to influence staff to implement EBP [20, 21]. Leadership knowledge is the theoretical or practical understanding of leadership that’s acquired through experience or education, skills refer to the use of this knowledge in execution or performance, and ability is inherent or developed quality that enable a person to perform leadership tasks effectively [22]. Implementation leadership behaviours refer to a leader’s observable methods of facilitating the EBP implementation and includes the ways in which a leader interacts with others [23]. Actions refer to the specific steps taken by a leader that demonstrates specific behaviours. For example, to show recognition for staff’s efforts to change (behaviour), leaders may publicly praise their staff or provide salary raises (actions).

The aim of this study was to develop a context-fit training program prototype to enhance the leadership competencies and behaviour of EBP implementation of Chinese unit nurse managers. We aimed to answer the following research questions:

1. What leadership behaviours do Chinese nurse managers believe are critical to facilitate implementation of EBP?
2. What modifications are needed to ensure the leadership behaviours accessible to Chinese nurse managers?
3. What actions are required to operationalize each behaviour?
4. What competencies are needed to develop these behaviours?
5. What strategies are suitable to deliver the implementation leadership training program to unit nurse managers in China?

## Methods

### Design

We employed a descriptive qualitative research design to answer the research questions and develop the training program prototype [24, 25]. We chose the qualitative descriptive design to acquire an in-depth understanding of the development of the content (i.e., implementation leadership behaviours, actions and competencies) and delivery strategies of the training program prototype from the perspective of knowledge users. We used an integrated knowledge translation (IKT) approach [26] to involve knowledge users in co-developing the prototype. Knowledge users included nursing managers from various leadership levels in a Chinese hospital who have knowledge of and understand Chinese culture, the nursing context and have the potential for implementing the training program. Using an IKT approach allowed us to 1) develop a training program prototype that is compatible with the hospital context in China, 2) integrate Chinese cultural values into the prototype and 3) ensure outputs meet the intended users' needs [26]. We reported the study methods and findings following the Guidance for reporting intervention development studies in health research [27] (See Supplementary file 1), and Consolidated Criteria for Reporting Qualitative Research [28] (see Supplementary file 2).

### Theoretical framework

The Ottawa Model of Implementation Leadership (O-MILe) is a theoretical model illustrating the knowledge and skills required for leaders to practice change-oriented, task-oriented and relations-oriented leadership behaviours to facilitate implementation of EBP for high-quality healthcare delivery and positive patient, provider and system outcomes [13]. The embedded mechanisms of O-MILe indicate that successful implementation requires leaders to understand site-specific evidence–practice gaps, implementation strategies, and how leadership influences planned change processes [13]. Leaders must also have the skills to prioritise change, set target goals and encourage staff to practice evidence-based care for improved outcomes [11]. The O-MILe framework informed the development of the training program prototype in the present study, described how leadership influences the implementation of EBP.

### Settings and participants

The participating hospital is in Changsha, the capital and most populous city of Hunan Province in South-Central China. The tertiary hospital is responsible for providing specialized health services, medical education and research. It has 2200 beds and 2400 health professionals that serves over 60 million people province wide. The hospital’s leadership at the top of the management system consists of one dean and five vice deans, all of whom are physicians. One vice dean oversees the nursing department, which is responsible for the overall management of the nursing staff. The nursing department consists of one nursing director (registered nurse) and two associate nursing directors (registered nurses). The associate nursing directors oversee six sector nurse managers (registered nurses) who are responsible for six nursing sectors, namely surgical, internal medicine, emergency and critical care, outpatient, operation room, and maternal and childcare sectors. Each of these has around 10 nursing units, totalling 62 nursing units (54 general wards and eight intensive care units), with 73 unit nurse managers (including associate nurse managers) and 1,470 nurses. See Supplementary file 3 for the hospital’s management structure.

The proposed training program targeted implementation leadership competencies and behaviours of unit nurse managers. We used purposeful sampling to include managers at different nursing management levels with diverse knowledge and experience, to ensure a comprehensive representation of perspectives and insights within nursing leadership and clinical nursing practice. The eligible criteria included individuals with: 1) formal managerial role at the time of recruitment (e.g. nurse director, sector nurse manager, unit nurse manager), 2) worked full-time at the hospital at the time of recruitment, 3) had over ten years of clinical nursing and at least five years of nursing management experience, 4) had previous experience conducting or participating in EBP implementation project, and 5) were able to speak, read and write in Chinese, willing to join the study, and able to give informed consent. The nursing director in the hospital distributed information about the study to all eligible nurse managers and asked them to contact the primary researcher if they were interested in participating.

### Training program development process

From November 2021 to March 2022, the primary researcher who is fluent in Chinese worked with the managers (*n* = 7) to codevelop the training program prototype. The development process encompassed four phases that involved four rounds of group discussions and three rounds of individual interviews with each manager (*n* = 21 interviews in total). The 4-phase development process is described below (also see Table 1). The managers were interviewed in between the group discussions to obtain in-depth reflections on developing content (i.e. behaviours, actions and competencies) and delivery strategies of training program, which were then summarised and discussed in the subsequent discussions. An open non-hierarchical format was used during the discussions so the managers and researcher could share power for making decisions regarding the training program design. Each discussion lasted 30–90 min, and the interview duration was 20–70 min. All the materials provided to the managers were translated into Chinese from their original format in English. The conformity of the materials was ensured through the back translation technique. The primary researcher and a professional translator with a health science background translated the materials independently and compared the Chinese and English version multiple times to ensure accuracy. All questions that were asked during the interview and discussions (see Table 2) were formulated in accordance with the O-MILe [13], and insights from previous O-MILe-based interventions [11, 29]. The evidence sources and development process of each element of the training program prototype are available in Supplementary file 4.

**Table 1:** Implementation leadership training program prototype development process and activities

Training program development process (Duration: 5 months)		Development activities
**Phase 1:** **Study introduction and preparation**	**Discussion I**	• Introduction of the study • Discussion on what leadership actions nurse managers need take to facilitate evidence-based practices (EBP) implementation • Described Ottawa Model of Implementation Leadership (O-MILe) and implementation leadership behaviours
**Phase 2:** **Training program content development**	**Interview I**	• Explored importance of leadership behaviours in O-MILe to implement EBP in Chinese nursing context • Revised behaviours to be understandable in Chinese nursing context • Selected actions corresponding to each leadership behaviour
	**Discussion II**	• Presented the aggregate feedback from interview I • Discussed and determine the importance rating and revisions of leadership behaviours • Discussed and determine actions corresponding to each leadership behaviour
	**Interview II**	• Presented and introduced tables of leadership behaviours, actions and examples of competencies • Explored competencies need to perform leadership behaviours and corresponded actions
	**Discussion III**	• Presented the aggregate feedback from interview II • Discussed and determined competencies need to perform each behaviour and action
Drafted template of the training program prototype		
**Phase 3:** **Training program delivery strategies development**	**Interview III**	• Introduced the content and the drafted training program prototype • Selected and proposed suitable delivery strategies or activities of training program
Modified the training program prototype		
**Phase 4:** **Training program prototype development**	**Discussion IV**	• Presented the new draft of training program prototype • Discussed and modified the training program prototype

*EBP* Evidence-based practice, *O-MILe* Ottawa Model of Implementation Leadership

**Table 2:** Interview and discussion questions

Training program development process (Duration: 5 months)		Questions
**Phase 1:** **Study introduction and preparation**	**Discussion I**	• What leadership actions should nurse managers take to facilitate the implementation of EBP?
**Phase 2:** **Training program content development**	**Interview I**	• How important is each leadership behavior in the O-MILe for nurse managers to implement EBP in Chinese nursing context? • How can we revise those behaviors to make them more understandable in the Chinese nursing context? • For each leadership behavior, what specific actions could managers take to effectively facilitate EBP implementation?
	**Discussion II**	• What are your thoughts on the importance ratings and revisions of leadership behaviors made in Interview I? Are there any additional modifications needed? • How do you evaluate the actions suggested in Interview I? Do you have any recommended modifications or other actions to consider?
	**Interview II**	• What competencies do nurse managers need to effectively perform each leadership behavior and its corresponding actions?
	**Discussion III**	• How do you perceive the competencies suggested in Interview I? • Are there any suggested modifications, or are there other critical competencies needed
Drafted template of the training program prototype		
**Phase 3:** **Training program delivery strategies development**	**Interview III**	• What strategies or activities do you think are appropriate for delivering the training program?
Modified the training program prototype		
**Phase 4:** **Training program prototype development**	**Discussion IV**	• What are your thoughts on the behaviors, actions, competencies, and delivery strategies we co-developed in previous interviews and discussions? • What modifications are needed to optimize the training program prototype? • Do you have any other suggestions to enhance the training program?

*EBP* Evidence-based practices, *O-MILe* Ottawa Model of Implementation Leadership

### Phase 1: study introduction and preparation

In the first group discussion, the researcher introduced the purpose of the training program, the managers’ roles in the development process and the importance of leadership in implementing EBP in clinical settings. The aim was to engage the managers in the training program development process, and ensure they understand the purpose of the co-development process. Then, managers discussed and listed the actions that unit nurse managers may take to promote the implementation of EBP. A scenario was provided to help them identify these actions. After that, the researcher introduced definitions of O-MILe and implementation leadership behaviours. Finally, the researcher presented the plan for developing the training program prototype to the managers and discussed it with them to refine it.

### Phase 2: training program content development

In the first round of interviews, the managers shared their perceptions and reasons for believing that the O-MILe leadership behaviours promote EBP implementation in the Chinese nursing context. A general five-point Likert scale was applied for knowledge users to rate the importance of each behaviour (1 = not at all important and 5 = very important). To ensure the O-MILe behaviours were applicable in China, managers were then asked if they had any suggestions for modifying them. Following this, the researcher presented a table containing examples of leadership actions and asked the managers to select, add or modify them to correspond to the O-MILe behaviours. Examples were based on suggestions given by the managers in the first discussion group and previous O-MILe interventions [11, 29].

In the second group discussion, the researcher presented an analysis of the first round of interviews with the managers. All seven managers discussed and then determined: 1) leadership behaviours critical for facilitating EBP implementation in the Chinese nursing context, 2) modifications required to those behaviours by Chinese nurse managers, and 3) actions that correspond to each leadership behaviour. Disagreements, ambiguities, and discrepancies were resolved through discussion.

After determining the actions that corresponded to each of the O-MILe leadership behaviours, managers participated in a second interview to understand the specific competencies they needed to operationalize each behaviour. The researcher introduced the modified leadership behaviours, their corresponding actions, and examples of competencies required to enact the behaviours. Examples of leadership competencies were summarised according to the O-MILe [13], and previous O-MILe-based interventions [11, 29]. Managers then identified the competencies needed for unit managers to perform the leadership behaviours and activities.

During the third discussion, the researcher presented the analysis from the second round of interviews and managers confirmed the competencies required for operationalizing implementation leadership. The competency items proposed by the managers were incorporated into tables.

### Phase 3: training program delivery strategy development

Following suggestions by the participating managers, the researcher drafted a template of the key elements and delivery strategies of the training program before the third interviews. The prototype was based on a recent systematic review of leadership interventions for nursing managers [30], the Template for Intervention Description and Replication (TIDieR) Checklist, and the content from Phase 2 of this study (i.e. leadership behaviours, actions and competencies). TIDieR is a 12-item checklist of intervention reporting elements [31]. Following the TIDieR checklist, the researcher drafted the elements in the template to describe: 1) the deliverers and participants of the training program (who); 2) materials and procedures (what); 3) modes of delivery, for example face to face, online, telephone (how); 4) locations for the training program to occur (where); and 5) duration and frequency of the modules (when and how much). In the template, content for each element was derived from our systematic review on the leadership development interventions for nurse managers [30], the O-MILe [13], and previous O-MILe-based interventions [11, 29]. During the interviews, the researcher introduced the training program content and the drafted template; managers then selected or proposed suitable delivery strategies or activities to be incorporated into the training.

### Phase 4: training program prototype development

In the fourth discussion, the researcher presented the training program prototype with managers’ ideas and reflections incorporated into it, and the prototype was further modified. Any disagreements, ambiguities or discrepancies among the researcher and managers were discussed until consensus was reached.

### Data analysis

Discussions and interviews were all in Chinese and audio-recorded and transcribed by two doctoral students whose first language is Chinese and who are fluent in English. We analysed the importance rating of the leadership behaviours with the Software Package Statistical Analysis (SPSS) Version 22.0 [32]. NVivo [33] was used to organise the data and facilitate analysis of qualitative data [34]. The data from the discussions and rounds of interviews were analysed immediately after each round, and the analysis results were sent to the participants for member checking [35, 36]. Analyses were conducted based on the thematic analysis approach described by Braun and Clarke [37]. First, the two researchers reviewed the transcripts several times to familiarise themselves with the data. Then, meaningful units of the transcribed text, such as paragraphs, sentences, and words, were inductively extracted to codes. Any differences in coding were discussed, and final codes were agreed upon and formed a codebook. After all the data were coded, the two researchers discussed and sorted the codes into potential themes and/or subthemes inductively, and collated the relevant data extracts. During this process, each new transcript was used to confirm or refute previously written codes and summary statements. The two independent researchers coded the data and reached consensus on codes or themes to enhance credibility. Finally, researchers reviewed and refined all themes together. The transcripts were analysed in Chinese, and the results were translated into English to be presented and discussed with the research team. The primary researcher and the professional English translator translated the results together. One of the research team members (fluent in both Chinese and English) read all materials to confirm the accuracy of the Chinese codes and interpretations.

### Trustworthiness

We utilized different strategies to ensure the trustworthiness (credibility, transferability, dependability, and confirmability) of study results [36]. To enhance the credibility of the results, we applied purposive sampling to recruit nurse managers who possessed extensive clinical nursing, nursing management, and EBP implementation experience. Two independent researchers conducted data analysis and discussed the findings to ensure the accuracy of the data analyzed. A third researcher reviewed the analysis process to maintain credibility. Furthermore, member checking was performed by all nurse managers to ensure the accuracy of the data. We maintained a codebook to ensure the dependability of the analysis process.

To ensure transferability and confirmability of the study findings, we provided detailed descriptions of study purpose, design, participants recruitment, prototype development, data collection and analysis methods, and the time period for each interview. This ensured that the study findings could be transferred to other settings or populations with similar characteristics. We also maintained detailed documentation to facilitate an external audit of our findings and to ensure confirmability.

### Ethical considerations

Ethical approval was obtained from the University Health Sciences and Sciences Research Ethics Board (No. H-01–20-5355) and the Institute Research Board of the participating hospital in China (No. 2019-S552). All seven participating managers provided their signed informed consent before participating in the development process.

## Results

Finally, seven managers in different nursing leadership roles and practice areas were invited and all agreed to participate in the study, including: associate director (*n* = 1), sector nurse managers (*n* = 2), and unit nurse managers (*n* = 4). The demographics of the seven managers was presented in Table 3.

**Table 3:** Demographic information of unit nurse managers (*n* = 7)

	Age	Current position	Education	Years of clinical nursing practice	Years of nursing management
1	44	Associate nursing director	Doctoral degree	26	15
2	47	Sector nurse manager	Master’s degree	26	17
3	43	Sector nurse manager	Master’s degree	26	10
4	47	Unit nurse manager	Master’s degree	28	15
5	42	Unit nurse manager	Master’s degree	22	5
6	44	Unit nurse manager	Master’s degree	26	14
7	45	Unit nurse manager	Master’s degree	27	18

### Perceived importance of implementation leadership behaviours in O-MILe

All the O-MILe behaviours were rated as important or very important lead implementation of EBP in the Chinese nursing context (see Table 4). Among these behaviours, all seven managers unanimously perceived nine of them as very important, accounting for 100% agreement. Six out of the seven managers considered two behaviours to be very important (86%), while five out of the seven managers rated one behaviour as highly important (71%). However, only one manager (14%) deemed the remaining two behaviours as very important, while the other six managers considered them important.

**Table 4:** Importance of leadership behaviours in O-MILe to facilitate the EBP implementation in Chinese nursing context n (%)

	**Leadership behaviours**	**Not at all important**	**Slightly important**	**Moderately important**	**Important**	**Very important**	**Reasons**
**Manager change behaviours**	Demonstrate commitment to change					7 (100%)	• Breaking doubts • Gaining support • Promoting involvement of nurses
	Understand and act on difficulties with change					7 (100%)	• Developing feasible plans • Promoting success implementation of EBP
	Reinforce vision and goals for change					7 (100%)	• Starting point for implementation of EBP • Being consistent • Motivating nurses • Improving work efficiency
	Advocate for change internally and externally					7 (100%)	• Creating supportive environment
**Manager relation behaviours**	Recognize efforts to change					7 (100%)	• Increasing nurses’ confidence • Promoting involvement of nurses
	Support change visibly and symbolically				1 (14%)	6 (86%)	• Addressing implementation barriers • Promoting involvement of nurses
	Facilitate interprofessional consensus on change				1 (14%)	6 (86%)	• Gaining recognition and supporting
	Communicate with staff about practice					7 (100%)	• Understanding concerns and difficulties • Promoting involvement of nurses • Developing feasible plans
**Manager task behaviours**	Clarify roles and responsibilities					7 (100%)	• Promoting duty execution
	Modify documentation forms				2 (29%)	5 (71%)	• Providing guidance
	Procure resources, education, and policies					7 (100%)	• Providing support
	Monitor performance and outcomes					7 (100%)	• Understanding implementation process • Making timely adjustment
	Provide reminders				6 (86%)	1 (14%)	• Promoting implementation of EBP • Decreasing wrongdoings
	Conduct regular leadership meetings to plan				6 (86%)	1 (14%)	• Providing guidance

*EBP* Evidence-based practice

Specifically, all the managers perceived the four change-oriented behaviours as very important because they would enable managers to address any doubts and gain the support of nurses, physicians and patients while developing implementation plans, creating supportive environments, and engaging nurses in the implementation process. For example, managers believed that nurses would become actively involved in the process of changing their practice based on evidence once they recognised the goals or potential benefits such as better patient outcomes from implementing EBP. In addition, Manager 4 stated that ‘*nurses, physicians or patients may question the implementation of EBP when they do not see any visible effects, especially at the beginning of implementation’* and managers need to show their determination to *‘break their doubts and keep the implementation going’*.

Similarly, more than half of managers believed that the relations-oriented behaviours (*n* = 4) were very important for boosting nurses’ confidence, understanding, and addressing nurses’ concerns and obstacles in addition to engaging nurses in EBP implementation and gaining recognition and support from interprofessional staff. Manager 6 claimed that ‘*unlike nurses of our generation who received more setback education (deliberately introduce a series of setbacks for trainees to encounter, to prepare them better to deal with these situations later in life), the young nurses nowadays need more recognition and encouragement’*, with Manager 2 recognizing ‘*the more they were encouraged, the better they performed’*. Manager 1 pointed out that communication mechanisms are critical to ‘*understand nurses’ feelings or difficulties in the implementation process’* and adjust the implementation plans accordingly.

Among the six task-oriented behaviours, most managers viewed clarifying roles, monitoring operations and performance, modifying nursing procedures and efficiently using resources as very important behaviours. By doing so, they could monitor the implementation process and make adjustments when needed, encourage individuals to accomplish tasks in the implementation team and seek more support (e.g. resources and policies) and guidance.

Two behaviours were rated as important (as opposed to very important) by six of the managers; these behaviours were: ‘provide reminders’ and ‘conduct regular leadership meetings to plan’. Managers admitted that providing reminders could encourage nurses and decrease the risk of forgetting to implement EBP, while ‘*once the EBP was embedded in the nursing procedures and nurses were fully engaged in implementing it, reminders would be like adding icing on the cake’* (Manager 5) because* ‘nurses are capable of completing any nursing work (without reminder)’* (Manager 7)*.* While managers confirmed that team meetings were important to reflect on and plan the implementation process, manager 7 stated that they ‘*should not have too many meetings because nurses and managers are already extremely busy in their daily work*’.

### Modifications in the O-MILe implementation leadership behaviours

As shown in Table 5, knowledge users confirmed that 10 of the 14 O-MILe behaviours were easily understandable and did not need modifications. Three others needed to be modified by adding clarifications, and the left one needed adapting to be culturally relevant. First, managers modified the behaviour from ‘Recognize efforts to change’ to ‘Recognize *ideas or actions* to change’ as they believed that managers should encourage nurses to express their ideas and engage them in EBP implementation processes. Second, ‘seek advice’ was added to the behaviour ‘Communicate with staff about practice’ since ‘*leaders should communicate with nurses about the EBP being implemented and also ask for their advice about the implementation*’ (Manager 5). Third, managers stated they conduct nursing team meetings to plan for the EBP implementation. Therefore, managers changed the O-MILe behaviour ' conduct regular *leadership* meetings’ to ‘conduct regular implementation team meetings’. Lastly, the O-MILe behaviour ‘Support change visibly and symbolically’, was changed to ‘Support change *substantively’* as symbolic support in Chinese was understood to mean there was no concrete substantive support.

**Table 5:**
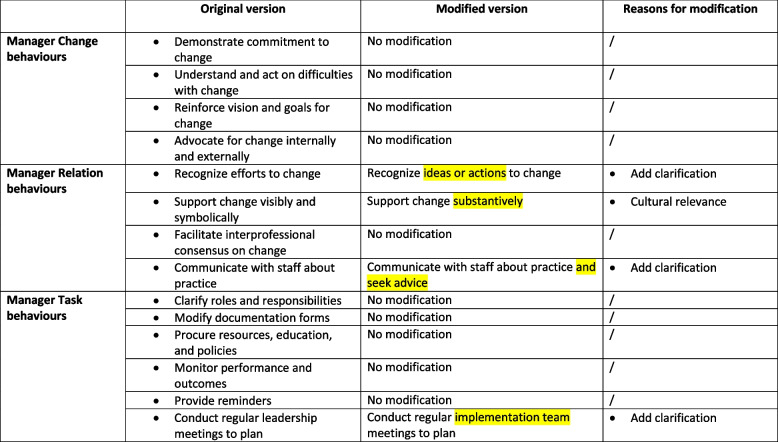
Modification of leadership behaviours in O-MILe

Modifications were highlighted in YELLOW

### Actions corresponding to implementation leadership behaviours

#### Actions for change-oriented behaviours

As shown in Table 6, participants suggested that unit nurse managers take the following actions to demonstrate their commitment to change: set goals, highlight the importance of EBP implementation, integrate EBP in nursing procedures, and report and analyse adverse events encountered in the implementation process. In addition, participants expressed the need for managers to participate in EBP implementation, discuss EBP implementation cases with nurses to identify barriers, and develop targeted implementation strategies. Explaining the short- and long-term benefits of EBP implementation was considered necessary to reinforce the vision and goals of the process. Showing nurses that the leadership supports EBP implementation at the hospital or nursing department level would also reinforce a vision. Managers also emphasized they could advocate for EBP implementation among patients, nurses, physicians and other interprofessional, hospital and nursing department-level leaders as well as external entities (e.g. nursing associations) via ways such as presenting the benefits of EBP implementation, and providing evidence-based health education. Publishing journal papers or presenting data on EBP at academic conferences were suggested as critical approaches to advocate for EBP implementation. Manager 4 stated, ‘*After I presented the outcome of 120 cases (patients) that experienced FTS (Fast Track Surgery, evidence-based perioperative interventions) in three national academic conferences, our director (physician) recognised the effect of FTS in facilitating postoperative recovery and asked all physicians to support the application of FTS*’.

**Table 6:** Competencies to develop specific implementation leadership behaviours and corresponding actions

Leadership behaviours in O-MILe		Actions corresponding to each behaviour	Competencies nurse managers need to perform each behaviour
**Change behaviours**	**Demonstrate commitment to change**	1. Set goals 2. Highlight importance of EBP implementation and push the implementation 3. Embed EBP into nursing procedures 4. Report and analyses adverse events	1. Skills to set goals for EBP implementation 2. Skills/abilities to integrate EBP implementation into unit procedures 3. Skills to sustain the EBP implementation
	**Understand and act on difficulties with change**	1. Identify barriers to EBP implementation 2. Develop targeted solutions for barriers and plans for EBP implementation	1. Skills to identify barriers and facilitators to EBP implementation 2. Skills to develop implementation strategies
	**Reinforce vision and goals for change**	1. State explicitly the leadership support 2. Implement EBP on small scales to reveal direct/short-term benefits 3. Explain the potential long-term benefits	1. Knowledge of potential benefits and outcomes of EBP implementation 2. Abilities to develop indicators to measure effectiveness of EBP implementation
	**Advocate for change internally and externally**	1. Advocate EBP implementation externally: 1) Present clinical cases, effectiveness data, academic achievements 2. Advocate EBP implementation among nurses: 1) Explain the significance and urgency of EBP implementation 2) Encourage new technologies or patents application, and academic papers writing 3) Conduct new technology competitions 3. Advocate EBP implementation among patients 1) conduct evidence-informed patient health education lectures (online or in-person), workshops, scenario simulations, peer support meetings	1. Skills to interpret and communicate direct benefits of EBP implementation 2. Abilities to translate EBP implementation data into academic outputs 3. Skills to nurture EBP implementation atmosphere 4. Skills to conduct evidence-informed health education 5. Abilities to organize EBP-relevant activities (e.g., seminar)
**Relation behaviours**	**Recognize ideas or actions to change**	1. Provide verbal praise or material reward 2. Assist with career development 3. Promote self-actualization	1. Knowledge of and skills to motivate nurses (e.g., value display, praise or reward) 2. Skills to empower others 3. Knowledge of nurses’ career development approaches 4. Skills to assist career development
	**Support change substantively**	1. Before EBP implementation: 1) Communicate with nurses to understand their attitude towards EBP 2) Recommend EBP-related resources (e.g., databases, papers, guidelines) to nurses 2. Create supportive environment during EBP implementation	1. Skills to resolve conflicts 2. Abilities to promote cooperation and improve team cohesion
	**Facilitate interprofessional consensus on change**	1. Show the validity of the evidence 2. Get support from leaders 3. Involve interdisciplinary professionals in the EBP implementation process	1. Skills to interpret and communicate direct benefits of EBP implementation 2. Skills to build EBP implementation team 3. Abilities to improve team execution
	**Communicate with staff about practice and seek advice**	1. Explain the significance and potential benefits of EBP implementation 2. Encourage nurses comment on current nursing practices and the evidence	1. Knowledge of current practice 2. Knowledge of potential benefits of EBP implementation
**Task behaviours**	**Clarify roles and responsibilities**	1. Build the EBP implementation team 2. Organize standard training for implementation team	1. Skills to build EBP implementation team 2. Skills and abilities to organize training
	**Modify documentation forms**	1. Embed EBP into nursing procedures 2. Prepare EBP implementation audit forms	1. Knowledge of indicators to measure the effectiveness of EBP implementation 2. Abilities to distribute task and supervise
	**Procure resources, education, and policies**	1. Provide education/training resources 2. Procure human resources and physical resources 3. Seek policy support	1. Abilities to acquire information (e.g., education/training opportunities) 2. Abilities to seek policy support
	**Monitor performance and outcomes**	1. Monitor implementation process 2. Set up reward and punishment regulations	1. Knowledge of indicators to measure the effectiveness of EBP implementation 2. Skills to audit and track the EBP implementation process and outcomes 3. Skills to sustain the EBP implementation
	**Provide reminders**	1. Nurse level: 1) Use oral reminder 2) Use reminder cards 3) Develop task schedule 2. Patient level: 1) Conduct on-one-on education 2) Use reminder cards 3) Use graphic education manuals or videos 4) Use health education account (e.g., in WeChat) 5) Conduct patient education meetings 6) Provide telephone reminders 7) Involve family members	1. Knowledge of and skills to conduct evidence-informed health education 2. Ability to learn and use new media
	**Conduct regular implementation team meetings to plan**	1. Conduct weekly/monthly implementation team meetings to discuss, develop, and modify implementation plans	1. Ability to organize meetings 2. Ability to develop plans

*EBP* Evidence-based practice

#### Actions for relations-oriented behaviours

Unit nurse managers can recognise nurses’ ideas or actions to change by providing verbal praise or material rewards, assisting nurses in their career development, and professionally empowering nurses. To this end, participants reported that they could help nurses identify their career goals and provide training opportunities to achieve them. The behaviour ‘Support change substantively’ included introducing EBP-related resources before implementation, and creating a supportive environment for nurses after initiating the implementation process. To facilitate interprofessional consensus, participants felt it was important to reveal the benefits of EBP implementation and gain senior leadership support. Involving interprofessional staff and increasing their understanding of the EBP implementation process was another way to reach a consensus identified by the managers. They considered communicating with nurses to include explaining the significance and benefits of EBP implementation as well as asking them for feedback.

#### Actions for task-oriented behaviours

Managers suggested that actions in the task-oriented behaviours involve building and training the EBP implementation team, adding EBP to nursing procedures and developing audit forms, providing training opportunities for EBP implementation, acquiring human and physical resources, seeking policy support from inside and outside the hospital that advance EBP implementation, monitoring the implementation process, setting up reward and punishment regulations, providing reminders to nurses and patients and conducting meetings to discuss, plan and adjust the implementation process. Notably, managers emphasised that the documents used should be refined when integrating EBP implementation into the existing nursing procedures. Manager 2 said ‘*the documents used such as audit form should be simple and applicable to clinical practice to avoid increasing nurses’ burden*’.

### Competencies that nurse managers need to perform leadership behaviours

Participants stated that unit nurse managers should have three categories of general competencies to develop the required implementation leadership behaviours: 1) knowledge of EBP in clinical nursing, and skills to identify clinical problems and EBP solutions such as searching, assessing and selecting evidence; 2) professional nursing knowledge and skills (e.g. clinical specialty, communication); 3) knowledge of implementation leadership. Specific competencies for developing different behaviours were also identified (see Table 6). For example, to develop change-oriented behaviours, managers need to have skills to set goals, identify barriers and develop implementation strategies, embed EBP in nursing procedures, and develop indicators to assess implementation, as well as knowledge of potential benefits of EBP and skills to interpret and communicate benefits, including academic outputs (e.g. journal papers). For relations-oriented behaviours, nurse managers noted that managers need to be familiar with current nursing practices and EBP, and be equipped with the skills and abilities needed to motivate others (e.g. assist career development), build an implementation team, resolve conflicts, and promote collaborations among team members. To develop task-oriented behaviours, they suggested that managers need skills to train the EBP implementation team, organise evidence-informed activities (e.g. health education and meetings), develop plans, monitor and sustain the implementation process and acquire information (e.g. relevant training opportunities), resources and policy support for advancing EBP implementation.

### Strategies to deliver the training program

Participating managers co-developed the strategies to deliver the training program with the researcher (Table 7). As suggested, the training sessions would involve twelve unit nurse managers, assigned to one of two groups and delivered by two experienced nurse managers and two researchers. The content would be delivered in-person through five modules in 4 days (total 32 h). Activities in the modules include lectures, experience sharing, group discussions, plan development, and coaching.

**Table 7:** Strategies to deliver the training program

Elements of training program prototype		Delivery strategies or activities
**Target participants**		• Units nurse managers (*n* = 12)
**Goals**		• To enhance implementation leadership behaviours of unit nurse managers and related knowledge, skills and abilities via facilitating implementation of EBP
**Objectives**		1. To increase basic knowledge and skills related to EBP 2. To discuss and develop essential nursing professional knowledge, skills and abilities needed for EBP implementation 3. To understand the importance of leadership in EBP implementation and the effective implementation leadership behaviours as described in OMILe 4. To improve knowledge, skills and abilities for developing each implementation leadership behaviour in OMILe 5. To be able to take actions for each leadership behaviour to facilitate EBP implementation
**Modules**		1. What should we know about EBP? 2. What are the essential professional knowledge, skills and abilities needed for EBP implementation? 3. Why leadership matters in EBP implementation and what are the effective implementation leadership behaviours? 4. What leadership knowledge, skills and abilities I need to develop for each implementation leadership behaviour? 5. What leadership actions I can apply to facilitate EBP implementation?
**Training program deliverers**	**Type, number & expertise**	• Four deliverers in total, including nursing researchers (*n* = 2), and nurse managers (*n* = 2). Nursing researchers should have research experience in nursing leadership and EBP implementation. Nurse managers should be those 1) co-developed the prototype, 2) have over 20 years of clinical nursing experience, and 3) over five years of nursing management experience, 4) have experience of conducting or participating in EBP implementation
	**Role**	• Role of deliverers: 1. Provide reading materials to participants before each module 2. Give lectures, share experience, and facilitate discussions during each module 3. Involve in the whole training process and provide (online/in-person) supervising for participants in or between each module 4. Follow up and coaching when needed 5. Provide research guidance (e.g., journal manuscript writing, revision, journal selection or submission) for participants who are willing to publish the EBP implementation process and results
	**Supplemental support**	• Supplemental support from the organizational nursing department, pursue support (e.g., training certificate or continuing education credits) from professional nursing association
**Duration of training program**		• Modules: approximately 32 h of full-time, in-person training in 4 consecutive days • Post-module activities: TBD
**Setting**		Places away from the hospital setting
**Pre-modules screening**		• Investigate evidence-based practice competencies (Evidence-based Practice Competence Questionnaire) of participants, invite participants with high levels of evidence-based practice competencies to give lecture in the Module 1 • Explore participants’ individual needs for intervention content and form, modify training program prototype accordingly
**Pre-module preparation**		• Each participant prepares 1–2 clinical nursing problems he/she currently encountering
**Arrangement of modules**	**Content of modules**	• Implementation leadership competencies, behaviours and actions
	**Activities for modules**	• lectures, experience sharing, group discussions, plans development, and coaching
**Post-module activities**		• Plans implementation, follow up, coaching, and final presentation day

*EBP* Evidence-based practice, *O-MILe* Ottawa Model of Implementation Leadership, *TBD* To be determined

Before the first module, the managers suggested that training participants should choose 1–2 clinical nursing problems they are currently encountering as the focus for each group. Post-module activities included undertaking plans, coaching, and a final presentation of their implementation. Notably, managers recommended that all modules should be delivered outside the hospital setting. As Manager 7 said, ‘*if (being trained) in the working environment, I will feel a sense of constraint … worry about phone calls’,* and Manager 3 added, *‘I feel relaxed and can concentrate on the training when I’m away from the hospital’*.

Managers proposed several ways to engage participants in the training program: 1) involve managers with high levels of EBP competencies to deliver content; 2) invite managers to participate with previous EBP implementation experience; and 3) provide guidance on writing academic paper, and journal selection for participants who are interested in publishing their EBP implementation. Managers emphasized that academic outputs are important when advocating for EBP implementation and for gaining support both from within and outside the hospital, and it is also one of the requirements for nurses’ promotion in management. Support from nursing researchers was described as important because ‘*few unit nurse managers receive standard research courses or training’* (Manager 1). Therefore, it was suggested that nursing researchers be included as deliverers of the training program with experienced managers to strengthen the content on research and emphasize the importance of EBP implementation.

### Prototype of the implementation leadership training program

Based on study findings and evidence-based resources, seven essential aspects of the training program prototype were identified:

1. Clarify the end goal of the training program—to develop competencies to enhance implementation leadership behaviours of unit nurse managers.
2. Deliver the program in two phases: in-person learning modules and post-module activities (i.e. follow up, coaching and final presentation).
3. The five learning modules corresponding to five objectives. Modules 1–3: general competencies (EBP; professional nursing; implementation leadership: see Table 5), Module 4: competencies for developing specific implementation leadership behaviours, Module 5: actions to operationalise each behaviour.
4. Engage nursing researchers (*n* = 2) with academic expertise and in-house managers (*n* = 2) with clinical expertise to co-deliver the program.
5. Include action learning processes for identifying clinical nursing problems; searching for, assessing and selecting evidence; and developing and undertaking a leadership action plan and EBP implementation plan. A leadership action plan specifies the actions managers need to undertake to support implementation and an implementation plan highlights how the evidence will be implemented on their unit.
6. Develop and conduct individualized development plans for enhancing their implementation leadership behaviours and competencies. The plans specify how to develop the competencies required for each manager to effectively enact implementation leadership behaviours, based on a self-rating of the Implementation Leadership Scale.

## Discussion

In this paper, we described our approach to working with seven nurse managers to co-develop a prototype for a training program based on the O-MILe conceptual framework. The study results indicated that the managers considered all the O-MILe leadership behaviours crucial for EBP implementation in their setting. They believed that most of the O-MILe behaviours were understandable, with only four of the 14 behaviours requiring some modification to add clarifications or cultural relevance. The managers suggested that the behaviours should be operationalized with actions directed towards nursing practices (e.g. EBP implementation), nurses (e.g. communicate, motivate, support), patients (e.g. educate), interprofessional staff such as physicians (e.g. involve in implementation process), incentives and resources (e.g. conduct EBP training), organization (e.g. pursue support from hospital or nursing department leaders, write EBP into regulations or policies) and external entities (e.g. presence at conferences). Notably, the managers identified the need to demonstrate the benefits of EBP implementation to key stakeholders, including point-of-care nurses, senior leaders, physicians, professional associations, as a crucial action. To enact these behaviours, findings from our study revealed that the managers agreed nurse managers require three types of general competencies 1) EBP, 2) professional nursing, and 3) implementation leadership. In addition, each type of behaviour and action requires specific competencies. Various methods such as involvement of in-house expertise were suggested to engage potential participants in the training program.

The nurse managers in the study acknowledged the importance of all implementation leadership behaviours in the O-MILe to promote EBP implementation in the Chinese nursing context. They believed that most of the behaviours were applicable in China and did not require any modification. These findings are consistent with a qualitative descriptive study by Cheng et al. [15] which examined the leadership practices of nurse managers in China. We found that Chinese nurse managers’ implementation leadership practices were congruent with those in Western countries such as Canada. In light of the cultural differences between Chinese and Western leadership practices, we propose several possible reasons for this congruency. First, the three types of implementation leadership behaviours in O-MILe (i.e. relations, change, and task-oriented behaviour) match some ideas of leadership in traditional Chinese culture. For example, the standards and rules emphasized in Legalism are closely linked to task-oriented leadership behaviours that focus on planning, controlling, problem-solving, and monitoring to efficiently achieve goals [38]. This is consistent with Ma et al.’s [39] study that claimed traditional Chinese philosophies are intertwined with Western leadership literature, and both influenced the current understanding of leadership in China. In addition, many of the principles and concepts underlying effective implementation leadership, such as relationship building, collaboration, and change management, are universally applicable and not limited to specific cultural contexts [15]. All nurse managers who participated in this study had previous experience with EBP implementation and recognized that the goal of EBP implementation is to improve the quality and efficiency of patient care. They understood the significance of exhibiting effective leadership behaviours to achieve this goal. For instance, managers emphasized the importance of relations-oriented leadership behaviours in boosting nurses’ confidence, understanding and addressing their concerns and obstacles, and engaging them in EBP implementation. This finding has been described across different countries or cultural contexts [40, 41].

Noteworthy, we found that the managers highlighted that the behaviour to ‘Communicate with staff about practice’ should be a two-way process, involving managers explaining EBP to point-of-care nurses while seeking advice from those nurses for better implementation. This two-way process seemed to contradict our original assumptions that managers in China have the sole authority to make decisions about EBP and nurses will do as they are told since they must obey and be loyal to senior authorities. While the Chinese culture does endorse a hierarchical working environment, it also values leaders who listen to the individual needs of followers to promote better task fulfilment and maintain organizational harmony [15]. Listening to individual needs aligns with the principles of Daoism which emphasizes that leaders should create harmony through respect and consideration for followers [42]. This was also true in other Eastern Asian countries. For instance, Yang and Horak [43] conducted a study in South Korea and discovered that despite the cultural emphasis on respecting hierarchical relationships and obedience to power, subordinates could vastly influence leaders’ decisions via formal and informal communication cannels. Therefore, when planning future interventions in those countries, it is critical to not only focus on the power of leaders in EBP implementation, but also to make sure that the voices of subordinates are heard and highly valued.

The managers who participated in our study suggested that unit nurse managers can take various actions to facilitate EBP implementation, implying that unit level nurse managers play multiple roles in the implementation process. For instance, participants in the present study proposed that managers could plan EBP implementation by setting implementation goals, identifying barriers, developing targeted solutions and plans, building and training the EBP implementation teams, and embedding EBP into nursing procedures. Similarly, Urquhart and colleagues [44] found that managers should identify the needs for implementation and then plan appropriately to ensure these needs are addressed. Additionally, similar to Boutcher et al. [45] and Giannitrapani et al. [46], managers in our study suggested that nurse managers could provide point-of-care nurses with training opportunities, acquire resources, seek policy support, refine documents used in EBP, and monitor the implementation process. Furthermore, participants proposed that nurse managers have a strong role in facilitating and motivating point-of-care nurses to implement EBP by providing verbal praise or material reward, assisting with career development, promoting self-actualization, communicating with nurses, and addressing inconsistencies or conflicts with EBP implementation. These findings align with previous studies by Birken et al. [47] and Engle et al. [48] which explicated the role of middle managers in the implementation of EBP. Identifying the roles and actions of unit level nurse managers is critical to better prepare them to lead the EBP implementation [49].

According to the managers in our study, showcasing the benefits of EBPs was the most frequent suggested action across different behaviours to facilitate the implementation process. Similarly, Birken et al. [6] revealed that showing the benefits to staff is an important way to encourage their participation in implementation of EBPs. Findings from our study found that not only is it an important action to influence point-of-care nurses, but also to facilitate interprofessional consensus, gain support from senior leaders at the hospital and external organizations such as professional nursing association. In our study, managers reported that they were frequently asked about the value of EBP by nurses and physicians when initiating implementation. Despite the increasing number of healthcare professionals in China who believe in the idea of establishing evidence-based clinical practices [2], many still worry that this approach may cause unnecessary changes in routine procedures, take up extra time, increase their workloads, be unacceptable to patients, or lead to no improvements or worsen patient outcomes [18, 50]. The managers in our study proposed alternative ways to showcase the benefits such as presenting effectiveness data, sharing clinical cases (e.g. nurses’ or patients’ experience of involving in EBPs implementation), and conducting evidence-informed education sessions. This findings is consistent across various contexts and highlights the need for unit nurse managers to act as knowledge broker to convince others of the importance and benefit of the EBP implementation [44, 45, 48].

### Strengths and limitations

Strengths of this study included the engagement of experienced unit level managers to co-develop the prototype. In addition, we provided a detailed description of the co-development process, including the evidence resources used for content and delivery strategy development, which allows for future replication by researchers.

Our study also has some limitations. First, we acknowledged that we only recruited seven managers and the small sampling size might limited the strength of the results. The COVID-19 pandemic occurred during the data collection process and limited the number of knowledge users we could involved in the interviews or discussions. We were not able to determine the saturation of the data. Second, the researcher participated in the discussions was also responsible for data analysis, which was valuable in terms of the training program development, but may provide a source of bias. We therefore include another researcher to perform the independent data analysis. Third, although a professional translator was involved in translating the development materials and study results, there is still a possibility of inaccurate translation, which could result in an incorrect interpretation of the findings.

### Contribution to literature

- Researchers could engage in-house expertise to assist with the development of context-specific training programs.
- Unit nurse managers could play multiple roles in EBP implementation, including planning, training, acquiring resources, seeking internal and external support, refining documents, motivating point-of-care nurses, coordinating and monitoring the implementation process.
- The identified implementation leadership competencies, behaviours and actions could be used to guide unit nurse managers to lead the EBP implementation in healthcare settings in China.
- Unit nurse managers could take various actions to showcase the benefits of EBP implementation to staff, interprofessional teams, senior leaders, and external organizations to gain support and promote consensus.

## Conclusions

We developed a training program prototype to enhance the implementation leadership behaviours of Chinese unit nurse managers, with the aim of promoting the implementation of EBP. Nurse managers identified all O-MILe leadership behaviours as essential for unit nurse managers to lead EBP implementation in China. This study revealed various leadership actions unit nurse managers can take to perform implementation leadership behaviour, as well as general and specific competencies required for developing these behaviours. Participating nurse managers also proposed strategies and activities that could be utilized to develop implementation leadership behaviours and competencies of unit nurse managers and facilitate EBP implementation in China. Our study implied that it is crucial for hospital administrators to ensure that unit nurse managers possess the required competencies related to EBP, professional nursing, and implementation leadership to lead EBP implementation. Policymakers and healthcare settings administrators should provide resources and support to assist unit nurse managers in developing implementation leadership competencies. Integrated knowledge translation can be a valuable approach for developing training programs that are relevant to a specific healthcare context. Further studies with large sample sizes from different clinical settings are needed to facilitate the generalization of the findings and gain an in-depth understanding of the program from different perspectives.

### Supplementary Information

Supplementary Material 1.

## Data Availability

The datasets used and/or analysed during the current study are available from the corresponding author on reasonable request.

## Abbreviations

EBP: Evidence-based practice
IKT: Integrated knowledge translation
O-MILe: Ottawa Model of Implementation Leadership
SPSS: Software Package Statistical Analysis
TIDieR: Template for Intervention Description and Replication
