# Supplementary material for: Development of a training program prototype to enhance implementation leadership competencies and behaviours of Chinese unit nurse managers: a qualitative descriptive study
Source: BMC Nurs. 2024 May 30;23:359. doi: 10.1186/s12912-024-01989-8 (PMC11137952; doi:10.1186/s12912-024-01989-8)
Supplement: Supplementary file 1 — Supplementary Material 1. [file 12912_2024_1989_MOESM1_ESM.docx]

**Supplementary file 1 GUIDED – a guideline for reporting for intervention development studies**

| **Item description** | **Explanation** | **Page in manuscript where item is located** | **Other*** |
| --- | --- | --- | --- |
| 1.Report the context for which the intervention was developed. | Understanding the context in which an intervention was developed informs readers about the suitability and transferability of the intervention to the context in which they are considering evaluating, adapting or using the intervention. Context here can include place, organisational and wider sociopolitical factors that may influence the development and/or delivery of the intervention (15). | Page 9-10 |  |
| 2.Report the purpose of the intervention development process. | Clearly describing the purpose of the intervention specifies what it sets out to achieve. The purpose may be informed by research priorities, for example those identified in systematic reviews, evidence gaps set out in practice guidance such as The National Institute for Health and Care Excellence or specific prioritisation exercises such as those undertaken with patients and practitioners through the James Lind Alliance. | Page 8-9 |  |
| 3. Report the target population for the intervention development process. | The target population is the population that will potentially benefit from the intervention – this may include patients, clinicians, and/or members of the public. If the target population is clearly described then readers will be able to understand the relevance of the intervention to their own research or practice. Health inequalities, gender and ethnicity are features of the target population that may be relevant to intervention development processes. | Page 8-9 |  |
| 4. Report how any published intervention development approach contributed to the development process | Many formal intervention development approaches exist and are used to guide the intervention development process (e.g. 6Squid (16) or The Person Based Approach to Intervention Development (17)). Where a formal intervention development approach is used, it is helpful to describe the process that was followed, including any deviations. More general approaches to intervention development also exist and have been categorised as follows (3): Target Population-centred intervention development; evidence and theory-based intervention development; partnership intervention development; implementation-based intervention development; efficacybased intervention development; step or phased-based intervention development; and intervention-specific intervention development (3). These approaches do not always have specific guidance that describe their use. Nevertheless, it is helpful to give a rich description of how any published approach was operationalised | Page 11-14 |  |
| 5. Report how evidence from different sources informed the intervention development process. | Intervention development is often based on published evidence and/or primary data that has been collected to inform the intervention development process. It is useful to describe and  reference all forms of evidence and data that have informed the development of the intervention because evidence bases can change rapidly, and to explain the manner in which the evidence and/or data was used. Understanding what evidence was and was not available at the time of intervention development can help readers to assess transferability to their current situation. | Page 11-14 |  |
| 6. Report how/if published theory informed the intervention development process. | Reporting whether and how theory informed the intervention development process aids the reader’s understanding of the theoretical rationale that underpins the intervention. Though not mentioned in the e-Delphi or consensus meeting, it became increasingly apparent through the development of our guidance that this theory item could relate to either existing published theory or programme theory | Page 9, 11-14 |  |
| 7. Report any use of components from an existing intervention in the current intervention development process | Some interventions are developed with components that have been adopted from existing interventions. Clearly identifying components that have been adopted or adapted and acknowledging their original source helps the reader to understand and distinguish between the novel and adopted components of the new intervention. | Page 11-14 |  |
| 8. Report any guiding principles, people or factors that were prioritised when making decisions during the intervention development process. | Reporting any guiding principles that governed the development of the application helps the reader to understand the authors’ reasoning behind the decisions that were made. These could include the examples of particular populations who views are being considered when designing the intervention, the modality that is viewed as being most appropriate, design features considered important for the target population, or the potential for the intervention to be scaled up. | Page 11-14 |  |
| 9. Report how stakeholders contributed to the intervention development process. | Potential stakeholders can include patient and community representatives, local and national policy makers, health care providers and those paying for or commissioning health care. Each of these groups may influence the intervention development process in different ways. Specifying how differing groups of stakeholders contributed to the intervention development process helps the reader to understand how stakeholders were involved and the degree of influence they had on the overall process. Further detail on how to integrate stakeholder contributions within intervention reporting are available (19). | Page 9, 11-14 |  |
| 10. Report how the intervention changed in content and format from the start of the intervention development process. | Intervention development is frequently an iterative process. The conclusion of the initial phase of intervention development does not necessarily mean that all uncertainties have been addressed. It is helpful to list remaining uncertainties such as the intervention intensity, mode of delivery, materials, procedures, or type of location that the intervention is most suitable for. This can guide other researchers to potential future  areas of research and practitioners about uncertainties relevant to their healthcare context. | Page 11-14 |  |
| 11. Report any changes to interventions required or likely to be required for subgroups. | Specifying any changes that the intervention development team perceive are required for the intervention to be delivered or tailored to specific sub groups enables readers to understand the applicability of the intervention to their target population or context. These changes could include changes to personnel delivering the intervention, to the content of the intervention, or to the mode of delivery of the intervention. | Page 19-20 |  |
| 12. Report important uncertainties at the end of the intervention development process | Intervention development is frequently an iterative process. The conclusion of the initial phase of intervention development does not necessarily mean that all uncertainties have been addressed. It is helpful to list remaining uncertainties such as the intervention intensity, mode of delivery, materials, procedures, or type of location that the intervention is most suitable for. This can guide other researchers to potential future areas of research and practitioners about uncertainties relevant to their healthcare context. | Page 23-24 |  |
| 13. Follow TIDieR guidance when describing the developed intervention. | Interventions have been poorly reported for a number of years. In response to this, internationally recognized guidance has been published to support the high quality reporting of health care? interventions5and public health interventions14. This guidance should therefore be followed when describing a developed intervention. | Page 21-25 |  |
| 14. Report the intervention development process in an open access format. | Unless reports of intervention development are available people considering using an intervention cannot understand the process that was undertaken and make a judgement about its appropriateness to their context. It also limits cumulative learning about intervention development methodology and observed consequences at later evaluation, translation and implementation stages. Reporting intervention development in an open access (Gold or Green) publishing format increases the accessibility and visibility of intervention development research and makes it more likely to be read and used. Potential platforms for open access publication of intervention development include open access journal publications, freely accessible funder reports or a study web-page that details the intervention development process. | / |  |

*e.g. if item is reported elsewhere, then the location of this information can be stated here.

**Supplementary file 2 Consolidated criteria for reporting qualitative studies (COREQ): 32-item checklist**

| **No. Item** | **Guide questions/description** | **Reported on Page #** |
| --- | --- | --- |
| **Domain 1: Research team and reﬂexivity** | | |
| *Personal Characteristics* |  |  |
| 1. Interviewer/facilitator | Which author/s conducted the interview or focus group? | Page 11 |
| 2. Credentials | What were the researcher’s credentials? E.g. PhD, MD | Page 16 |
| 3. Occupation | What was their occupation at the time of the study? | Page 16 |
| 4. Gender | Was the researcher male or female? | Male, page 11 |
| 5. Experience and training | What experience or training did the researcher have? | Page 16 |
| *Relationship with participants* |  |  |
| 6. Relationship established | Was a relationship established prior to study commencement? | No, page 10 |
| 7. Participant knowledge of the interviewer | What did the participants know about the researcher? e.g. personal goals, reasons for doing the research | Page 11 |
| 8. Interviewer characteristics | What characteristics were reported about the interviewer/facilitator? e.g. Bias, assumptions, reasons and interests in the research topic | Page 16 |
| **Domain 2: study design** | | |
| *Theoretical framework* |  |  |
| 9. Methodological orientation and Theory | What methodological orientation was stated to underpin the study? e.g. grounded theory, discourse analysis, ethnography, phenomenology, content analysis | Page 9 |
| *Participant selection* |  |  |
| 10. Sampling | How were participants selected? e.g. purposive, convenience, consecutive, snowball | Page 10 |
| 11. Method of approach | How were participants approached? e.g. face-to-face, telephone, mail, email | Page 11 |
| 12. Sample size | How many participants were in the study? | Page 10 |
| 13. Non-participation | How many people refused to participate or dropped out? Reasons? | None |
| *Setting* |  |  |
| 14. Setting of data collection | Where was the data collected? e.g. home, clinic, workplace | Page 10  . |
| 15. Presence of non-participants | Was anyone else present besides the participants and researchers? | None |
| 16. Description of sample | What are the important characteristics of the sample? e.g. demographic data, date | Page 11 |
| *Data collection* |  |  |
| 17. Interview guide | Were questions, prompts, guides provided by the authors? Was it pilot tested? | Yes, page 11-17 |
| 18. Repeat interviews | Were repeat interviews carried out? If yes, how many? | No |
| 19. Audio/visual recording | Did the research use audio or visual recording to collect the data? | Page 15 |
| 20. Field notes | Were ﬁeld notes made during and/or after the interview or focus group? | None |
| 21. Duration | What was the duration of the interviews or focus group? | Page 11 |
| 22. Data saturation | Was data saturation discussed? | Yes, page 30 |
| 23. Transcripts returned | Were transcripts returned to participants for comment and/or correction? | No |
| **Domain 3: analysis and ﬁndings** | | |
| *Data analysis* |  |  |
| 24. Number of data coders | How many data coders coded the data? | Page 15 |
| 25. Description of the coding tree | Did authors provide a description of the coding tree? | No |
| 26. Derivation of themes | Were themes identiﬁed in advance or derived from the data? | Page 15 |
| 27. Software | What software, if applicable, was used to manage the data? | Page 15 |
| 28. Participant checking | Did participants provide feedback on the ﬁndings? | Page 15 |
| *Reporting* |  |  |
| 29. Quotations presented | Were participant quotations presented to illustrate the themes/ﬁndings? Was each quotation identiﬁed? e.g. participant number | Yes, page 17-24 |
| 30. Data and ﬁndings consistent | Was there consistency between the data presented and the ﬁndings? | Yes, page 25-26 |
| 31. Clarity of major themes | Were major themes clearly presented in the ﬁndings? | Yes, page 17-25 |
| 32. Clarity of minor themes | Is there a description of diverse cases or discussion of minor themes? | Yes, page 17-25 |


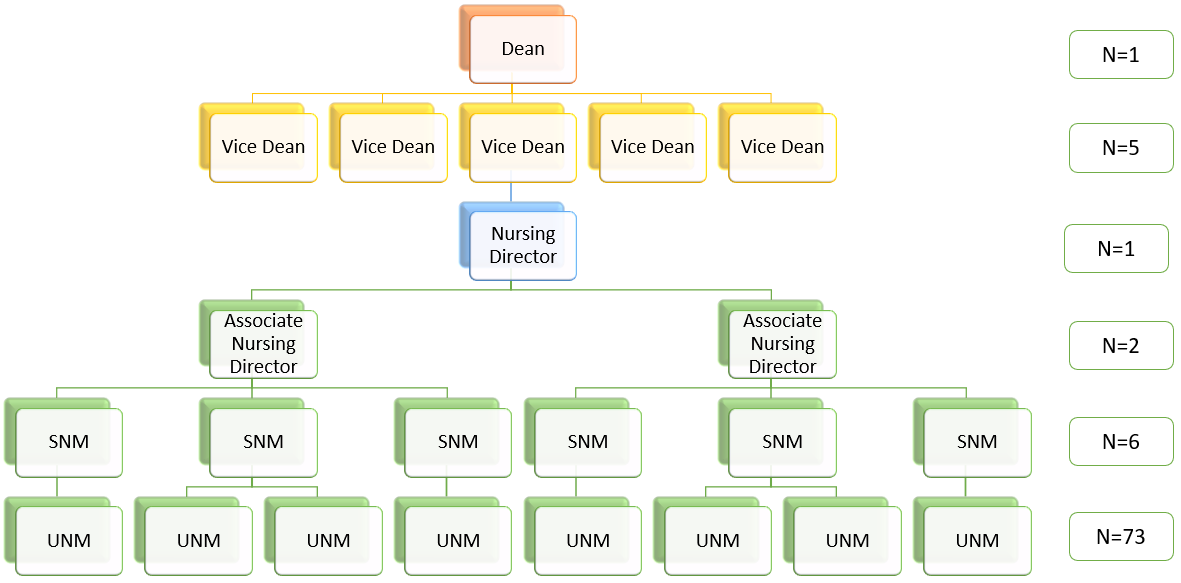
**Supplementary file 3 Hierarchical nursing management structure in the participating hospital**

**Notes**: SNM: Sector Nurse Manager; UNM: Unit Nurse Manager

**Supplementary file 4 Evidence sources and development process for elements of training program prototype**

| **Elements of training program prototype** | | **Evidence sources** | | **Development process** | | | | | | |
| --- | --- | --- | --- | --- | --- | --- | --- | --- | --- | --- |
|  |  | **Systematic review** (30) | **O-MILe/ Previous O-MILe interventions**  (11,13,29) | **Phase 1** | **Phase 2** | | | | **Phase 3** | **Phase 4** |
|  |  |  |  | **Discussion1** | **Interview 1** | **Discussion2** | **Interview 2** | **Discussion 3** | **Interview 3** | **discussion 4** |
| **Target recipients** | |  | √ | √ |  |  |  |  | √ | √ |
| **Goals** | |  | √ | √ |  |  |  |  | √ | √ |
| **Objectives & Modules** | |  | √ |  | √ | √ | √ | √ |  |  |
| **Training program deliverers** | **Type, number & expertise** |  | √ |  |  |  |  |  | √ | √ |
|  | **Role** | √ | √ |  |  |  |  |  | √ | √ |
|  | **Supplemental support** | √ |  |  |  |  |  |  | √ | √ |
| **Duration of the training program** | |  | √ |  |  |  |  |  | √ | √ |
| **Setting** | |  |  |  |  |  |  |  | √ | √ |
| **Pre-module activities** | | √ | √ |  |  |  |  |  | √ | √ |
| **Arrangement of modules** | **Content of modules** |  | √ |  | √ | √ | √ | √ | √ | √ |
|  | **Activities for modules** | √ | √ |  |  |  |  |  | √ | √ |
|  | **Duration of modules** |  | √ |  |  |  |  |  | √ | √ |
| **Post-module activities** | | √ | √ |  |  |  |  |  | √ | √ |

**Note:** O-MILe, Ottawa Model of Implementation Leadership.
